# 5-HTR3 and 5-HTR4 located on the mitochondrial membrane and functionally regulated mitochondrial functions

**DOI:** 10.1038/srep37336

**Published:** 2016-11-22

**Authors:** Qingyi Wang, Huiyuan Zhang, Hao Xu, Dongqing Guo, Hui Shi, Yuan Li, Weiwei Zhang, Yuchun Gu

**Affiliations:** 1 Laboratory of Molecular Pharmacology, Institute of Molecular Medicine, Peking University, Beijing, China

## Abstract

5-HT has been reported to possess significant effects on cardiac activities, but activation of 5-HTR on the cell membrane failed to illustrate the controversial cardiac reaction. Because 5-HT constantly comes across the cell membrane via 5-HT transporter (5-HTT) into the cytoplasm, whether 5-HTR is functional present on the cellular organelles is unknown. Here we show 5-HTR3 and 5-HTR4 were located in cardiac mitochondria, and regulated mitochondrial activities and cellular functions. Knock down 5-HTR3 and 5-HTR4 in neonatal cardiomyocytes resulted in significant increase of cell damage in response to hypoxia, and also led to alternation in heart beating. Activation of 5-HTR4 attenuated mitochondrial Ca^2+^ uptake under the both normoxic and hypoxic conditions, whereas 5-HTR3 augmented Ca^2+^ uptake only under hypoxia. 5-HTR3 and 5-HTR4 exerted the opposite effects on the mitochondrial respiration: 5-HTR3 increased RCR (respiration control ratio), but 5-HTR4 reduced RCR. Moreover, activation of 5-HTR3 and 5-HTR4 both significantly inhibited the opening of mPTP. Our results provided the first evidence that 5-HTR as a GPCR and an ion channel, functionally expressed in mitochondria and participated in the mitochondria function and regulation to maintain homeostasis of mitochondrial [Ca^2+^], ROS, and ATP generation efficiency in cardiomyocytes in response to stress and O_2_ tension.

**5-HT** is also named serotonin and found even in fungi and plants in addition to animals^1^. Serotonin (5-hydroxytrptamine, 5-HT) in blood is released by enterochromaffine cells and is also synthesized in neurons and endothelial cells^2^. 5-HT exerts its physiological effects through 5-HT receptors which are a group of G protein-coupled receptors (GPCRs) and ligand-gated ion channels. They are classified to be 7 subfamilies (5-HT_1_ to 5-HT_7_), each coded by a separate gene. 5-HT3 is a ligand-gated ion channel, while all other serotonin receptors are GPCRs which activate an intracellular second messenger cAMP to produce excitatory or inhibitory effects. Despite modulation of serotonin at synapses receives a major attention in challenging depression, cardiovascular pathogenesis has also been addressed to associate with serotonin. Alternation in the heart rates^3^, a hypertrophic mechanism in cardiacmyocytes^4^ and parkinsonian heart^5^ lead to controversial suspicion and indistinct understanding in the role of 5-HT and its relative mechanisms in the heart. In mammalian cardiovascular system, 5-HT3 receptor is believed to be located on afferent vagal and sympathetic neurons, which mediate reflex bradycardia and pain^6^, while 5-HT4 receptors are found to be present in cardiac atria and ventricles^7^.

Heart is one of the most active tissues because its main function is to continuously pump blood to other tissues. This continuous work requires a stable operation to provide energy in a relatively constant speed. It is well known that mitochondrion is a membrane-enclosed organelle, which generates most of the cell’s supply of energy in the form of adenosine triphosphate (ATP)^8^. In addition to ATP generation, mitochondrial Ca^2+^ flux plays important roles in many other cellular activities, including cytoplasmic Ca^2+^ signals and activation of cell death pathways^9^. Mitochondrial Ca^2+^ was uptaken through a Ca^2+^ -selective ion channel- mitochondrial Ca^2+^ uniporter (MCU) driven by voltage across the inner mitochondrial membrane (IMM)^10^. This electrochemical gradient is generated by proton pumping the respiration chain. Under pathological conditions such as ischemia-reperfusion, the increases in cytosolic Ca^2+^ concentration leads to mitochondrial Ca^2+^ accumulation and myocardial injury. This accumulation is achieved by activation of mitochondrial Ca^2+^ uniporter during ischemia. The mitochondrial Ca^2+^ overload results in opening a nonspecific pore in the inner mitochondrial membrane, mitochondrial permeability transition pore (mPTP), which is permeable to small molecules. Opening of mPTP usually occurs at early reperfusion. The cell death mediators such as apoptosis inducing factor (AIF) and cytochrome c are released to cytosol followed by mPTP opening^11^. Increase in inhibition to mPTP opening caused by reperfusion injury provides an obvious target for cardioprotection.

The previous researches has shown the evidence that 5-HT transporter (5-HTT) is expressed in cardiac cells, and the environmental 5-HT concentration affects the heart cells^12^. Besides 5-HT effect through its receptors on the cell membrane^13^,^14^, the mechanism which we unveiled highlighted another novel path: 5-HT can directly activate its functional receptors on the mitochondrial membrane, and regulate mitochondrial and cellular activities and functions.

We first exploited the location of 5-HTR in the cardiac mitochondria. Semi-quantification PCR revealed that 5-HTR3 and 5-HTR4 were present in both rat and mice hearts (data not shown). 5-HTR3 and 5-HTR4 were shown to be localized on the membrane of cardiomyocyte mitochondria, by co-immunostaining with an inner-mitochondria membrane marker, COX VI (Fig. 1A). These co-localizations were further confirmed by Western Blot results (Fig. 1B). The purity of mitochondria was controlled by using SERCA2 and Lamin which are expressed on the reticulum and nuclear membrane respectively. In new-natal cardiomyocytes, Ca^2+^ transient was used to represent the myocyte contraction. Cardiomyocytes of which 5-HTR3 and 5-HTR4 were knocked down by siRNA (37 ± 2.23 min^−1^) exhibited an increase of 59.14% in the contraction frequency comparing with the control group (23.25 ± 1.49 min^−1^) (Fig. 1C).

ATP, mostly produced in mitochondria, decreased rapidly in cytoplasm after a cell injury or oxygen depletion. As indicated in Fig. 1D, under the normoxic condition, ATP content in 5-HTR3 and 5-HTR4 knocking down cardiomyocytes was not significantly different from the control groups. However, after cultured in anoxic environment for 12 hours, 5-HTR3 and 5-HTR4 knock-down cardiomyocytes exhibited a significant decrease (12.04%) in intracellular ATP content. Knock down 5-HTR3 or 5-HTR4 alone respectively did not result in any significant changes in intracellular ATP content under both normoxic and hypoxic conditions.

Lactate dehydrogenase (LDH), a biomarker of cellular damage, was measured under the condition of normoxia and hypoxia. In normoxic condition, 5-HTR3 and 5-HTR4 knock-down cardiomyocytes showed a slight increase (6.76%) in the release of LDH into the culture medium (Fig. 1E). After culture the cells in a hypoxic condition, 5-HTR3 and 5-HTR4 knock-down cardiomyocytes showed a significant elevation (38.48%) in LDH release. Knock down 5-HTR3 or 5-HTR4 alone respectively did not result in any significant changes in LDH release under both normoxic and hypoxic conditions.

Under the normal condition, high concentration of Ca^2+^ could stimulate mitochondrial Ca^2+^ uptake (MCU), thus stimulate mitochondrial bioenergetics (NADH production) to generate ATP^15^. Serotonin hydrochloride (5-HT) as well as serotonin creatinine sulfate at the concentration of 100 μM significantly reduced 42.13779% and 35.07% of mitochondrial Ca^2+^ uptake respectively (Fig. 2A,B,C), suggesting that 5-HT receptors on mitochondria affected its functions. Moreover, this Ca^2+^ uptake in the mitochondria was mediated by a ruthenium red (RuR)-sensitive mechanism (Fig. 2D). Inhibition of mitochondrial Ca^2+^ uptake by RuR was in a dose-dependent manner.

The across mitochondrial membrane potential (∆Ψ) serves as the driving force of mitochondrial Ca^2+^ uptake. No difference in ∆Ψ between the groups of control and 5-HT was detected by the membrane potential sensitive fluorescent dye (Rh123) (Fig. 2E), suggesting that 5-HT reduced mitochondrial Ca^2+^ uptake independently of mitochondrial membrane potential.

5-HT is the general agonist for both 5-HTR3 and 5-HTR4. To clarify the distinct contribution of 5-HTR3 and 5-HTR4 in this inhibitory effect on mitochondria Ca^2+^ uptake, specific agonists and antagonists were further employed. Zacopride, a 5-HTR4 agonist^16^, attenuated mitochondrial Ca^2+^ uptake by 45.1677% (Fig. 3A,B), whereas m-chlorophenylbiguanide, a 5-HTR3 agonist, exerted no effect (Fig. 3E). The activation of 5-HTR4 induced mitochondrial Ca^2+^ uptake reduction was also confirmed by another 5-HTR4 agonist, BIMU (Fig. 3C). Moreover, RS 23597-190 hydrochloride, a 5-HTR4 antagonist partly reversed 5-HT inhibitory effect by 75.9055% (Fig. 3C). 5-HTR4 is a G-protein coupled receptor, which activates the cAMP-dependent pathway by activating adenylate cyclase (AC). 5-HT, zacopride, and BIMU significantly increased cAMP levels by 619.69%, 4484.01%, and 5622.68% respectively (Fig. 3B), in mouse cardiac mitochondria after incubated for 10 minutes. SQ 22536, an AC inhibitor, reversed the activation of 5-HTR4 by BIMU induced inhibition on Ca^2+^ uptake by 98.13461% (Fig. 3C,D), further suggesting this inhibitory effect is majorly mediated by 5-HTR4 via the Gαs pathway.

Surprisingly, the effect of 5-HT on mitochondrial Ca^2+^ uptake under hypoxic condition was opposite to that under the normoxia condition. 3 minutes for hypoxic challenge were employed in most cases as results shown in Fig. 3, 5-HT did enhance mitochondrial Ca^2+^ uptake by 87.37357% (Fig. 3F,G). M-chlorophenylbiguanide, a 5-HTR3 agonist, increased mitochondrial Ca^2+^ uptake by 31.47068% (Fig. 3G). However, the inhibition of Ca^2+^ uptake caused by 5-HTR4 under hypoxia was consistent with that under the normoxia. It reduced 52.08713% of mitochondrial Ca^2+^ uptake by zacopride (Fig. 3G). These results suggested that 5-HTR4 did not contribute to the augment effect of 5-HT on mitochondrial Ca^2+^ uptake under hypoxia, but 5-HTR3 were involved in.

5-HTR3 belong to the family of ligand-gate cation channels composed of five identical or homologous subunits^17^. In Fig. 3H, single channel currents from mitoplasts derived from cardiomyocytes were recorded at −100 mV. Under the control condition, no single-channel event could be observed. The application of 10 uM 5-HT activated discrete single-channel events of about 1 pA in patches from isolated heart mitoplasts (p < 0.01). As a response to 5-HTR3A antagonist, 3-AQC, the open probability of inward current was significantly reduced (p < 0.01) (Fig. 3J), suggesting that currents was mediated by 5HTR3^18^ by its unitary conductance and pharmacological sensitivity.

The rates of state 3 and state 4 respirations, and the Respiration Control Rate (RCR) were measured because they display the efficiency of the movement of electrons along the electron transport chain and the coupling of this movement to the production of ATP by oxidative phosphorylation. State 3 respiration rates were determined by the addition of ADP, and state 4 respiration was measured in the presence of adequate substrate but without addition of ADP. Mitochondrial rate of respiration was measured using an O_2_-sensing electrode in the cardiac mitochondria with or without the presence of 5-HTR3 agonists including m-Chlorophenylbiguanide hydrochloride and 1-Phenylbiguanide hydrochloride (Fig. 4). As shown in Fig. 4A, RCRs in mitochondria pre-incubated with 5-HTR3 agonists above were significantly higher than that in control groups (53.4538% and 29.6462%, respectively).

5-HTR4 was activated by different 5-HTR4 agonists respectively, including zacopride, ML 10302 Hydrochloride and BIMU (Fig. 4D). RCRs of mitochondria in which 5-HTR4 was activated, were lower than those in control groups (Fig. 4D). Reduction of RCR caused by Zacopride or ML 10302 Hydrochloride was significant in a dosage dependent manner (Fig. 4B,C).

The opening of mPTP would lead to uncouple oxidative phosphorylation and causing mitochondrial swelling. A high Ca^2+^ pulse is a widely accepted method to open mPTP, resulting in mitochondrial swollen. The swollen of mitochondria could be reflected by the decrease of relative UV-light absorbance. CsA, a common mPTP blocker, is therefore shown to prevent this swollen. Either 2 uM CsA or 5-HT3R agonists including 5-HT, m-Chlorophenylbiguanide hydrochloride and 1-Phenylbiguanide hydrochloride prevented mitochondrial swelling followed by a Ca^2+^ pulse of 200 uM for 60 s (Fig. 5A,B). (P < 0.05). Inhibitory effect by m-Chlorophenylbiguanide, 1-Phenylbiguanide, and 5-HT on Ca^2+^ induced mitochondrial swelling at 300 s and 500 s was shown in Fig. 5B.

5-HTR4 agonist (BIMU), exhibited a similar effect as CsA in preventing 200 uM Ca^2+^ induced mitochondrial swelling (Fig. 5B,C). Moreover, inhibition of AC, which is the downstream signal transduction consequent to activation of 5-HTR4, significantly stopped 200 uM Ca^2+^ induced mitochondrial swelling. These data are in strong support of the conclusion that 5-HTR4 protects mitochondria via Gαs pathway by attenuating the Ca^2+^ uptake and thus, reduce the mPTP opening probability.

5-HT actively comes across the cell membrane via 5-HT transporter (5-HTT) into the cytoplasm^12^. Regulation of intra-mitochondrial Ca^2+^ homeostasis is critical in both physiological and pathological functioning of the heart, resulting in the rate of energy production^19^, arrhythmia^20^,^21^,^22^, and even cell death^20^,^21^. Consistent to previous reports^22^,^23^,^24^, ruthenium red sensitive MCU accounted upon the majority of Ca^2+^ uptake in our study. 5-HT, as the general agonist for all sub categories of 5-HTR, inhibited mitochondria Ca^2+^ uptake and O_2_ consumption under normoxia, but enhanced mitochondria Ca^2+^ uptake and O_2_ consumption under hypoxia. Key steps of mitochondrial metabolism in ATP production are Ca^2+^ dependent^19^, it is therefore speculated to keep maintenance of ATP production in a relative constant speed by 5-HT under different O_2_ tension. Additionally, ROS is also recognized as an important signal molecule^25^ and homeostasis of ROS signaling was achieved by 5-HT effect on O_2_ consumption. By the way, it is well documented that hypoxia induces the elevation of intracellular Ca^2+^ via a variety of mechanisms^26^,^27^ including opening of voltage-gated channels, TRP channels, Ca^2+^ release from internal store^28^. Our results is consistent with previous reports that small increases in mitochondrial [Ca^2+^] takes place followed by a cytosolic [Ca^2+^] increase induced by hypoxia in cardiomyocytes^29^,^30^. Augment mitochondria Ca^2+^ uptake under hypoxia effectively buffers this elevation in intracellular Ca^2+^, and it limits the damage caused by Ca^2+^, especially in ischemia-reperfusion.

Due to mitochondrial and nuclear genes mutations, arrhythmia is often associated with patients of mitochondrial diseases^31^,^32^,^33^. Although abnormality of [Ca^2+^] in mitochondria buffering is well known to be involved in arrhythmia, the mechanisms are not clear^34^,^35^. It was known that mitochondria uptakes Ca^2+^ rapidly and buffer cytosolic [Ca^2+^] during E-C coupling, which supports the theory that fluxes in cytosolic [Ca^2+^] directly lead the increases in mitochondrial [Ca^2+^]. In 2006, Maack reported his findings that the role of mitochondrial Ca^2+^ buffering in cardiac myocytes during contraction is to match the energy/ATP production with demand. Our data supported their conclusion. As shown in Fig. 1D, in the condition of normoxia, ATP concentration remains constant in cardiomyocytes of control and 5-HTR3, 5-HTR4 knock-down groups. Since mitochondrial [Ca^2+^] increased is a signal to couple energy production in mitochondria when the lack in ATP supply, the larger frequency of cytosolic [Ca^2+^] transient in 5-HTR3, 5-HTR4 knock-down cardiomyocytes might be due to the maintenance of ATP level in cells. The alternations in frequency of cytosolic [Ca^2+^] transient accompanies with alternations in heart beating, further relating to arrhythmia.

5-HTR3 and 5-HTR4 are both functionally located on the mitochondrial membrane and exhibit distinct effects on the mitochondrial function. 5-HTR4 is a type of G-protein coupled receptors via Gαs subunits to generate cAMP as the second messenger^17^. 5-HT and 5-HTR4 agonists could significantly increase the concentration of cAMP in isolated heart mitochondria. Consistently, different agonists of 5-HTR4 displayed the similar effect and blockage of AC reversed the effect of activation of 5-HTR4, resulting in credibility of 5-HTR4 function. 5-HTR4 is shown important for maintaining the constant intra-mitochondrial Ca^2+^ concentration and lowers the risk of Ca^2+^ overload by reducing Ca^2+^ influx into mitochondria. 5-HTR3 is the only a ligand-gated ion channels in all 5-HTR families. It is noticed that 5-HTR3 exhibited only augment effect in mitochondria Ca^2+^ uptake while under hypoxia, suggesting the other second messengers are required in addition to activation of 5-HTR3.

The inhibition of mPTP opening induced by 5-HTR3 and 5-HTR4 could be physiologically important to protect mitochondria from loss of mitochondrial membrane integrity, and ability of generation of ATP. In addition, it could also reduce the pro-apoptotic proteins released from mitochondria and protect the heart from apoptosis. Heart is a high energy demanding organ. Shortage in O_2_ and substrate e.g. ischemia causes the dysfunction and even damage of cardiomyocytes. There is therefore a constant status for cardiomyocytes to keep homeostasis of mitochondrial [Ca^2+^], ROS, and ATP generation efficiency. Served as “guards”, 5-HTR3 and 5-HTR4 together protected cardiomyocytes by increasing the mitochondrial Ca^2+^ uptake when facing anoxia, which kept [Ca^2+^] homeostasis in the cytoplasm. At the same time, they cooperated to reduce the mPTP opening caused by increased [Ca^2+^] in mitochondria. As a result, the damage of ischemia/reperfusion injury on cardiomyocytes was minimized. 5-HT via its receptors 5HTR3 and 5-HTR4 on the mitochondria membrane contributes to this homeostasis. Our data contributes to a better understanding in how 5-HT, as a neurotransmitter and a potent activator in the blood stream, participates in the regulation of cardiac behaviors and how cardiomyocytes preform in response to O_2_ tension by using 5-HT in mediating its mitochondrial activities to pass through the stress.

## Methods and Materials

### Animal experiments

All of the animal experiments were approved by the Ethics Review Board for Animal Studies of IMM, Peking University. All methods were performed in accordance with the relevant guidelines and regulations.

### Mitochondria isolation and mitoplast preparation

Cardiac mitochondria were isolated from BALB/c mice by using a differential centrifugation method that retains mitochondrial structure and functions such as respiration and Ca^2+^ uptake^36^,^37^. After thoracotomy, heart were rapidly excised into an ice-cold isolation buffer (210 mM annitol, 70 mM sucrose, 5 mM HEPES, 1 mM EGTA, 0.5% BSA, pH7.2 adjusted with KOH), and washed 3 times. Atria and fat were removed and heart was chopped into small pieces. Heart was homogenized using glass homogenizer. The samples were the centrifuged for 10 min at 1000 g at 4 °C. The supernatant containing the mitochondria fraction was further centrifuged at 10000 g for 10 min at 4 °C (Centrifuge 5417 R, Eppendrof). Finally, the pellet was re-suspended in the isolation buffer and the protein concentration was determined. Samples were stored on ice before experiments and would be used up within 4 hours after isolation.

Mitoplast preparation was previously introduced by Kirichok *et al*. with modifications^24^. In order to obtain mitoplasts, the isolated mitochondria were subject to osmotic shock for 5 min in hypotonic solution consisting 5 mM sucrose, 5 mM HEPES and 1 mM EGTA (pH 7.2 with KOH). The sample was centrifuged for 5 min at 3920 g and subsequently re-suspended in the solution containing 750 mM KCl, 100 mM HEPES and 1 mM EGTA (pH 7.2 with KOH). The isolated mitoplasts were stored on ice before experiments and would be used up within 4 hours after isolation.

### Mitochondrial respiration measurements

Mitochondrial suspensions were diluted to a total volume of 1 ml in measurement buffer (220 mM manitol, 70 mM sucrose, 2 mM HEPES, 5 mM KH_2_PO_4_, 2.5 mM MgCl_2_, 0.5 mM EDTA, 1% BSA, pH 7.4 adjusted with KOH). The oxygen consumption rate was measured using an O_2_-sensing electrode -mitocell miniature respirometer MT 200 (Harvard Apparatus, Holiston, MA 01746, USA) at the room temperature. After recording state 4 oxygen consumption, 100 mM ADP was added to the chamber to induce state 3 respirations using microliter fixed-needle syringes.

### Mitochondrial [Ca^2+^] measurements

Mitochondrial Calcium concentration [Ca^2+^]_mito_ was probed by using Calcium indicator Quest Fluo-8^TM^, AM (AAT Bioquest, Sunnyvale, CA 94085, USA). Mitochondrial membrane potential was measured using Rhodamine 123 (Invitrogen/Molecular Probes, Carlsbad, CA, USA). Isolated mitochondria were suspended in potassium chloride (KCl) media (125 mM KCl, 2 mM K_2_HPO_4_, 1 mM MgCl_2_, 20 mM Hepes, pH 7.0) containing 5 mM succinate. Freshly prepared mitochondria were incubated with 5 uM Fluo-8 or Rhodmine123 for 15 min at room temperature, and observed using Nikon ECLIPSE Ti-u. After recording the baseline of fluorescence intensity, the mitochondria were treated with 10 uM Ca^2+^ or 5 uM FCCP (Sigma-Aldrich, St. Louis, MO, US). Both Fluo-8 and Rhodmine were excited at 488 and emissions were collected at 505–503 nm.

### Mitochondrial protein extraction

The mitochondria used for protein extraction was purified using LS Columns (Miltenyi Biotec, Auburn, CA 95602, USA). Mitochondrial pellet was dissolve in lysis buffer (1 ml RIPA, 10 ul PMSF) containing phosphatase inhibitor cocktail tablet (Roche Diagnostics GmbH, Mannheim, Germany), and Protease Inhibitor Cocktail Tablets (Roche Diagnostics GmbH, Mannheim, Germany). The samples were then centrifuged for 10 min at 12000 g at 4 °C. The supernatant containing mitochondrial protein was collected. Protein concentrations were measured by Bio-Rad Dc Protein Assay (Bio-Rad, Hercules, CA 94547, USA) using BSA as a standard according to the manufacturer’s instructions. The mitochondrial protein was stored at −80 °C until used.

### Western Blot analysis

The denatured protein was subjected to 12% Bis-Tris Gel followed by transferring to 0.45 uM PVDF membranes. Membranes were blocked with 5% nonfat milk in PBS for 1 h at room temperature and probed overnight with an anti-5-HTR4 (1:1000) (Santa Cruz Biotechnology, INC., Santa Cruz, CA, USA), anti-5-HTR3 (1:500) (Santa Cruz Biotechnology, INC., Santa Cruz, CA, USA), anti-COX IV (Cell Signaling, MA, USA), LaminA/C (Abcam (Hong Kong) Ltd., HKAP, New Territories, HK), Serca2a (Abcam (Hong Kong) Ltd., HKAP, New Territories, HK) antibody. Membranes were rinsed 3 times and incubated with secondary antibody for 2 hours.

### Semi-quantification PCR

After cDNA are synthesized using TransScript First-Strand cDNA Synthesis SuperMix kit (Beijin Transgen Biotech Co., LTD, Beijin, China), PCR was performed in 20 uL reaction mixture consisting of 7 uL dd H2O, 10 uL EasyTag Mix (Beijin Transgen Biotech Co., LTD, Beijin, China), 2 uL of each primer, and 1 uL cDNA. Primers used for 5-htr3 is 5′-ACCGCCTGTAGCCTTGAC-3′, and 5-htr4 is 5′-CCTTCTACATCCCGTTTC-3′. PCR was performed using an initial denaturation at 95 °C for 5 minutes, followed by 30 cycles at 95 °C for 40 seconds, 63.5 °C for 40 seconds, and 72 °C for 30 seconds. After 30 cycles, an additional elongation step was performed at 72 °C for 10 minutes. Amplified PCR products were run on 1.0% agarose gels containing ethidium bromide.

### Co-immunofluorenscence

Mitochondria centrifuged onto glass coverslips were fixed with freshly made paraformaldehyde (2%) in PBS for 20 min at room temperature. After rinsed 3 times, mitochondria were permeabilized with PBS containing 0.1% Triton X-100 for 5 min. The samples were blocked with 2% BSA in PBS. The coverslips were probed overnight with combination of an anti-5-HTR4 (1:100) and anti-COX IV antibody, or anti-5-HTR3 (1:100) (Santa Cruz Biotechnology, INC., Santa Cruz, CA, USA) and anti-COX IV (1:750) (Cell Signaling Technology, Danvers, MA, USA) Technology antibody, at 4 °C. After washing away the primary antibodies, the coverslips were incubated with combinations of secondary antibodies with two different wavelengths: anti-goat Texas red (1:500) and FITC anti-rabbit (1:500). The samples were rinsed and observed using Nikon N-SIM microscopy.

### Mitochondria swelling measurement

Mitochondria swelling and opening of mitochondrial permeability transition pore (mPTP) were measured by studying the rate of absorbance change at 520 nM using UV-vis Spectrophotomiter (UV-1750, Shimadzu) connected to a computer. Incubation conditions and inductions of mPTP opening were indicated in the legends of figures and results.

### Cell Culture

Neonatal mouse ventricular cardiomyocytes were cultured as previously introduced by Iwaki *et al*. with modifications^38^. Cardiomyocytes were isolated from 1- to 3-day-old BALB/c mice. Hearts removed and rapidly trisected and digested with pancreatin (0.1%, Gibco) and collagenase type II (0.03%, Worthington) for 6 minutes at 37 °C. The cell was collected from supernatant after centrifugation (1000 rpm, 5 minutes), and the pellet was resuspended in the buffer containing pancreatin and collagenase. The above steps were repeated for several times until the tissue was fully digested. The myocytes were cultured in medium containing Bulbecco’s modified Eagle’s medium (DMEM, Gibico) with fetal bovine serum and penicillin/streptomycin (100 ug/ml, Gibco). 8 × 10^6^ cells were plated on 100-mm plastic culture plate. The cells were cultured 24 hours before transfection. Physiological level 4 uM of 5-HT was added to medium 24 hours prior to the experiment.

### siRNA

siRNA against 5Htr3a and 5Htr4 were designed and purchased from GenePharma Inc.(Beijing, PR China). Three separated siRNA were designed against distinct regions of each gene and the knock-down efficiency was measured by Semi-quantification PCR and Western Blot. The ones with best efficiency were used for following experiments. siRNA against 5Htr3a is 5′-GAGGUGAAGUUCAGAACUATT-3′, scrambled is: 5′-GGGUUAGCACGAAAUAAGUTT-3′.

siRNA against 5Htr4 is CUGGCCUAUUACCGAAUCUTT-3′, scrambled is 5′-GCGUACUCACUGCAUCUAUTT-3′

### Lactate dehydrogenase (LDH) and Intracellular ATP content measurements

LDH released to cultured medium was quantified by colorimetric measurements using Lactate Dehydrogenase detection kit (Shanghai Gensource Co. Ltd., Shanghai, P.R. China) according to the manufacturer’s instructions. Extinctions of cells in different groups were measured by mirotiter plate reader (Promega Corporation, Madison, USA) at 570 nm. The intencity values were normalized to control for time points correspondingly.

CellTiter-Glo Luminescent Cell Viability Assay (Promega Corporation, Madison, USA) was used according to the manufacturer’s instructions. Luminescence signal was measured using synergy HT fluorometer (Bio Tek, Winooski, VT, USA). The values were normalized to control for each corresponding point. LDH releases from cardiomyocytes into the cultured medium and intracellular ATP content were analyzed after cultured in a hypoxic condition at 37 °C for 12 hours in comparison to normoxic groups.

### Measurement of spontaneous sytocolic [Ca^2+^] transients

Cells were loaded with 2 uM Quest Fluo-8TM, AM (AAT Bioquest, Sunnyvale, CA 94085, USA). The spontaneous sytocolic [Ca^2+^] transients were measured in Tyrode’s Buffer at ~35 °C, using a laser Laser Spinning Disk Confocal Microscope for Live Cell (Olympus, Japan).

### cAMP assay

Isolated heart mitochondria were lysed in 0.1 N HCl solution and cAMP was measured using a competitive binding ELISA (R&D System, Minneapolis, MN, US) according to the manufacturer’s protocol. For screening of pharmacologic response of 5-HTR4, mitochondria were pre-treated with 10 uM Serotonin hydrochloride (Sigma-Aldrich, St. Louis, MO, US), 10 uM BIMU (Tocris Bioscience, Bristol, UK), and 100 uM zacopride respectively (Tocris Bioscience, Bristol, UK).

### Electrophysiology study

Channel function was measured at room temperature by patch-clamp electrophysiology of mitoplast in the single-channel as previously introduced by Kirichok *et al*. with modifications^24^. All single-channel recordings were made in the cell-attach configuration of the patch-clamp technique. The bath solution consisted with 50 mM Na-gluconate, 10 mM HEPES, 1 mM EGTA and 1 mM EDTA (pH 7.2 with NaOH). Patch electrodes were filled with 150 mM CsOH, 5 mM CsCl, 135 mM sucrose, 10 mM HEPES, 1.5 mM EGTA and 1.5 mM EDTA (pH 7.2 with d-gluconic acid). Current was measured using Axon CNS digidata 1440 A, filtered at 1 kHz and sampled at 5 kHz.

## Additional Information

**How to cite this article**: Wang, Q. *et al*. 5-HTR3 and 5-HTR4 located on the mitochondrial membrane and functionally regulated mitochondrial functions. *Sci. Rep*. **6**, 37336; doi: 10.1038/srep37336 (2016).

**Publisher’s note:** Springer Nature remains neutral with regard to jurisdictional claims in published maps and institutional affiliations.

## Figures and Tables

**Figure 1:**
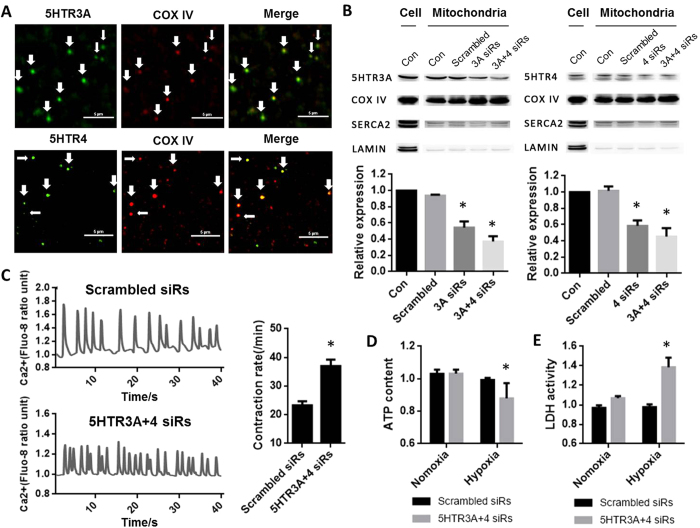
5-HTR3 and 5-HTR4 were present on mitochondria of Cardiomyocytes, and knock-down 5-HTR3 and 5-HTR3 significant resulting in increase of contract frequency, change of ATP content and LDH release. (**A**) Co-immunofluorescence analysis of localization of 5-HTR3 and 5-HTR4 respectively in cardiomyocyte mitochondria in which COX IV is a marker. The mitochondria were isolated from BALB/c mice. (a) co-immunofluorescence of isolated cardiomyocyte mitochondria, fixed and reacted with 5-HTR3 antibody (green) and COX IV antibody (red). Merge: the image shows 5-HTR3 expression in cardiomyocyte mitochondria. (b) Co-immunofluorescence of 5-HTR4 and mitochondrial marker COX IV. The immunoreactions show 5-HTR4 expression (green) in mitochondria (red). Calibration bar, 5 um. (**B**) Western Blot analysis confirmed the present of 5-HTR3 and 5-HTR4 on the cardiac mitochondria. COX IV was used as a biomarker of mitochondria, SERCA2a was the marker for ER, Lamin A/C was the maker of nucleus. (**C**) Knock-down 5-HTR3 and 5-HTR4 triggers significant increase of contract frequency reflected by intracellular Ca^2+^ transient in cultured neonatal mouse cardiomyocytes. Intracellular Ca^2+^ transient trace of (a) control myocytes (n = 10) and (b) myocytes knock down 5-HTR3 and 5-HTR4 after 3 day of siRNA transfer (n = 22) (c) Frequency of the spontaneous [Ca^2+^] transients. The data represent the means ± S.E. of n experiments from at least 3 cardiomyocytes. (**D**) Effect of 5-HTR3 and 5-HTR4 on intracellular ATP content of neonatal mouse cardiac myocytes under normoxic and hypoxic conditions. (**E**) Effect of 5-HTR3 and 5-HTR4 on LDH release of neonatal mouse cardiac myocytes under normoxic and hypoxic conditions. LDH release from cardiomyocytes into the cultured medium and intracellular ATP content were analyzed after cultured in a hypoxic condition at 37 °C for 12 hours in comparison to normoxic groups. Data is present as the mean ± SE of LDH group (n = 12), ATP group (n = 16).

**Figure 2:**
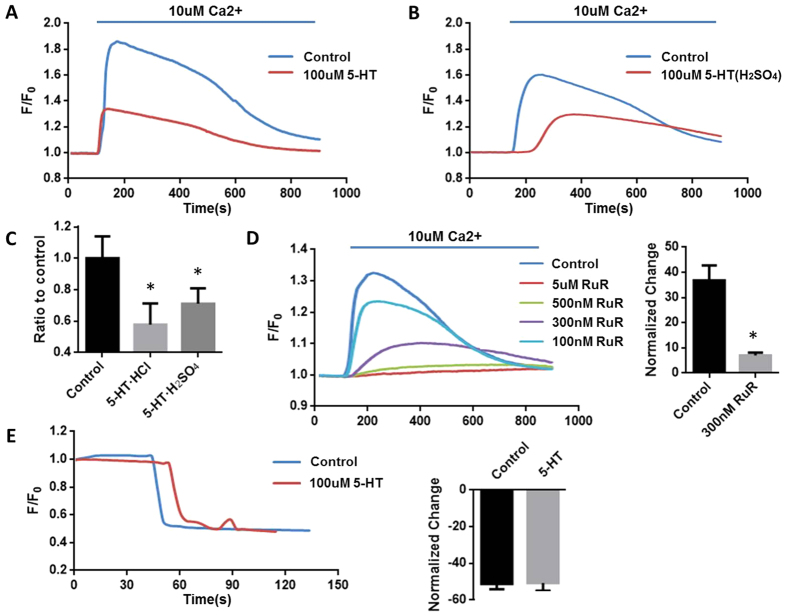
5-HT will attenuate Ca^2+^ uptake under the normoxia condition. The fresh-made mitochondria were pre-incubated with Calcium indicator Quest Fluo-8^TM^, AM or membrane potential dye Rhodamine 123 for 15 min and washed three times using potassium chloride (KCl) media containing 5 mM succinate. After recording the baseline, the mitochondria were perfused with 10 uM Ca^2+^ or 5 uM FCCP. (**A**) Serotonin hydrochloride (5-HT) decreases mitochondrial Ca^2+^ uptake. (**B**) Serotonin creatinine sulfate (5-HT(H_2_SO_4_)) attenuate Ca^2+^ uptake. (**C**) The percentage of Ca^2+^ uptaken by mitochondria of 5-HT groups (n = 6) and 5-HT (H_2_SO_4_) groups (n = 6). (**D**) Ca^2+^ induced mitochondrial Ca^2+^ uptake can be inhibited by RUR (n = 5). (**E**) Transmitochondrial potential (measured with 5 nM Rh-123) in control and 100 uM 5-HT pre-incubated mitochondria. 5 uM FCCP (Protonophore trifluoromethoxy carbonyl cyanide phenylhydrazond) was added to depolarize the mitochondria (n = 3).

**Figure 3:**
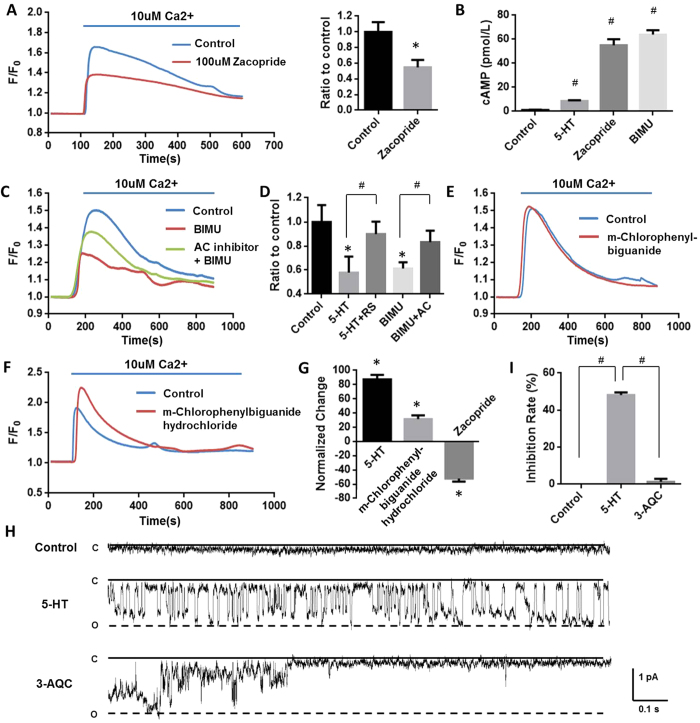
5-HTR4 reduced mitochondrial Ca^2+^ uptake under normoxia and 5-HTR3 augmented mitochondrial Ca^2+^ uptake under hypoxia. The fresh made mitochondria were perfused with oxygen-free medium for 3 min. After this, the mitochondria were replaced in oxygen-contained medium and the experiment was started immediately. (**A**) Zacopride (100 uM), a 5-HT receptors 4 agonist, attenuated mitochondrial Ca^2+^ uptake. n = 6 (P = 0.0267). (**B**) Effects of 5-HT, zacopride, and BIMU on cAMP levels in mouse cardic mitochondria after 10 minutes of treatment (p < 0.001 compared with controls, n = 6). (**C**) SQ22536 (AC inhibitor) partially reversed attenuating of Ca^2+^ uptake caused by BIMU, an agonist of 5-HTR4 (**D**) RS 23597-190 hydrochloride (RS), a 5-HTR4 antagonist, also reversed the inhibition of mitochondrial Ca^2+^ uptake by 5-HT. n > 6 at each column. (**E**) m-Chlorophenylbiguanide, a 5-HTR3 agonist, exerted no effect mitochondrial Ca^2+^ uptake (n = 5). (**F**) m-Chlorophenylbiguanide hydrochloride, a 5-HTR3 agonist, augmented Ca^2+^ uptake under the hypoxia condition. (**G**) Under hypoxia, 5-HT and m-Chlorophenylbiguanide hydrochloride augmented Ca^2+^ uptake whereas zacopride attenuated Ca^2+^ uptake, n > 8 at each group. (**H**), (**J**) Single channel currents induced by 5-HT, obtained from excised membrane from mitoplast of mice heart and inhibited by 3-AQC (n = 6). Single-channel recordings were made in the cell-attach configuration of the patch-clamp technique.

**Figure 4:**
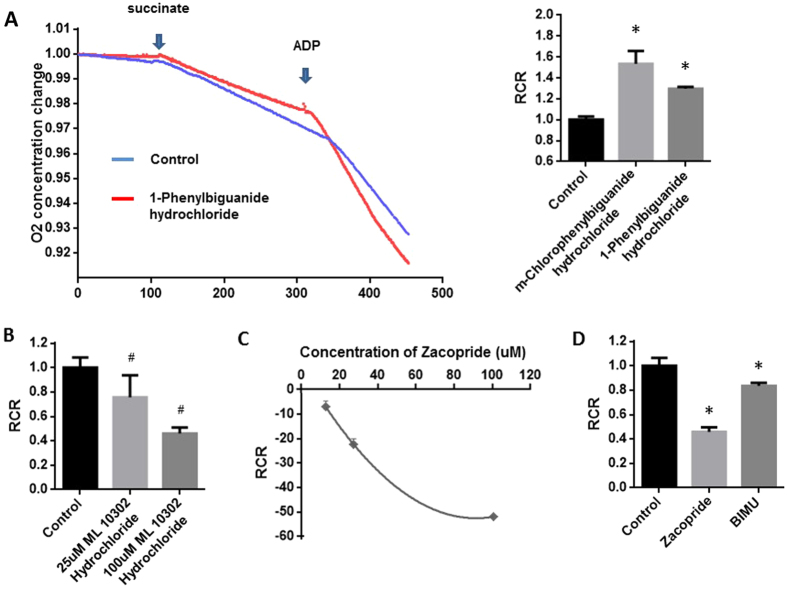
Augment effect of 5-HTR3 in contrast to inhibitory effect of 5-HTR4 on ADP-induced acceleration of mitochondrial respiration. Succinate and ADP were added at the indicated time point. Respiratory control ratio (RCR) was calculated as the ratio of state 3 to state 4 respiration rates. (**A**) Both m-Chlorophenylbiguanide hydrochloride and 1-Phenylbiguanide hydrochloride, an agonists of 5-HTR3, significantly increased RCR. n > 7 at each column (P < 0.05); (**B**) ML 10302 hydrochloride, an agonist of 5-HTR4, significantly inhibited RCR at the concentration of 25 μM and 100 μM, n > 6 at each column; (**C**) Zacopride, an agonist of 5-HTR4, reduced RCR in a dosage dependent manner, n > 7 at each points; (**D**) Zacopride and BIMU attenuated RCR, n > 8 at each column. Relative solvent was used as the vehicle control.

**Figure 5:**
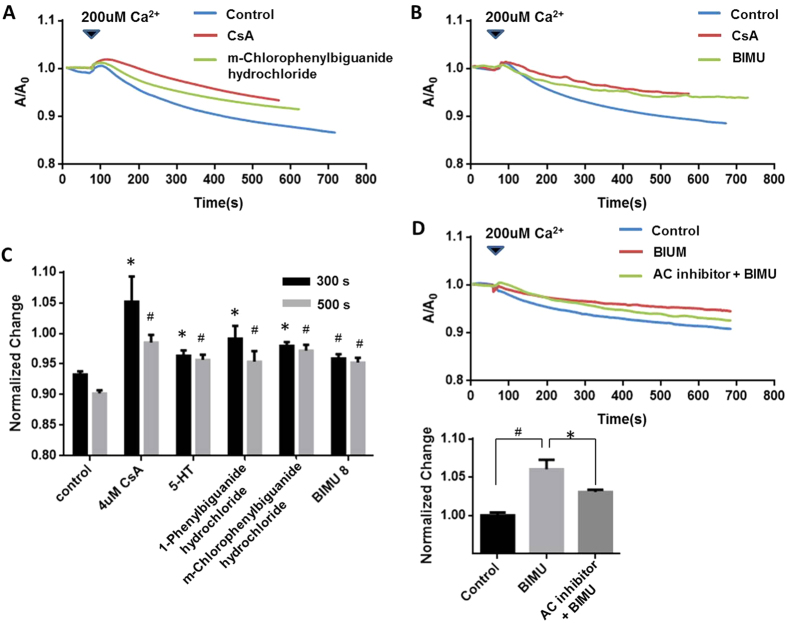
Both 5-HTR3 and 5-HTR4 inhibited mPTP opening reflected by the swollen measurement. For each measurement, the ice-cold mitochondria were incubated with medium alone, 5-HTR3/5-HTR4 agonists, or CsA at room temperature for 10 min prior to the experiments. 200 uM of Ca2+ was added at 60 s with a quick mix. (**A**) CsA and m-Chlorophenylbiguanide hydrochloride (5-HTR3 agonist) attenuated the mPTP opening; (**B**) BIMU attenuated the mPTP opening; (**C**) Inhibitory effects of 5-HTR3 and 5-HTR4 on mPTP opening. The bars represents the A/Ao at 300 s and 500 s of each experimental groups respectively, n > 8 at each column. (**D**) AC inhibitor overcame the effect on mPTP opening caused by BIMU at 500 seconds.
